# Hemophagocytosis, hyper-inflammatory responses, and multiple organ damages in COVID-19-associated hyperferritinemia

**DOI:** 10.1007/s00277-021-04735-1

**Published:** 2021-12-04

**Authors:** Guiying Dong, Jianbo Yu, Weibo Gao, Wei Guo, Jihong Zhu, Tianbing Wang

**Affiliations:** 1 Trauma Center, Key Laboratory of Trauma and Neural Regeneration (Peking University), Ministry of Education, Peking University People’s Hospital, No.11 Xizhimen South Street, Beijing, 100044 People’s Republic of China; 2 Emergency Department, Peking University People’s Hospital, No.11 Xizhimen South Street, Beijing, 100044 People’s Republic of China

**Keywords:** COVID-19, Hyperferritinemia, Hemophagocytic lymphohistiocytosis, In-hospital mortality

## Abstract

Hyperferritinemia comes to light frequently in general practice. However, the characteristics of COVID-19-associated hyperferritinemia and the relationship with the prognosis were not well described. The retrospective study included 268 documented COVID-19 patients. They were divided into the hyperferritinemia group (≥ 500 µg/L) and the non-hyperferritinemia group (< 500 µg/L). The prevalence of fever and thrombocytopenia and the proportion of patients with mechanical ventilator support and in-hospital death were much higher in the hyperferritinemia group (*P* < 0.001). The hyperferritinemia patients showed higher median IL-6, D-dimer, and hsCRP (*P* < 0.001) and lowered FIB level (*P* = 0.036). The hyperferritinemia group had a higher proportion of patients with AKI, ARDS, and CSAC (*P* < 0.001). According to the multivariate analysis, age, chronic pulmonary disease, and hyperferritinemia were found to be significant independent predictors for in-hospital mortality [HR 1.041 (95% CI 1.015–1.068), *P* = 0.002; HR 0.427 (95% CI 0.206–0.882), *P* = 0.022; HR 6.176 (95% CI 2.447–15.587), *P* < 0.001, respectively]. The AUROC curve was 0.88, with a cut-off value of ≥ 971 µg/L. COVID-19 patients with hyperferritinemia had a high proportion of organ dysfunction, were more likely to show hyper-inflammation, progressed to hemophagocytic lymphohistiocytosis, and indicated a higher proportion of death.

**Supplementary Information:**

The online version contains supplementary material available at 10.1007/s00277-021-04735-1.

## Introduction

COVID-19 caused by SARS-CoV-2 has become a pandemic worldwide [1, 2]. Although assertive China has weathered the “Darkest Hour” of COVID-19, the other countries are currently facing COVID-19 resurgence, entering the following waves of the pandemic [3].

Serum ferritin levels typically reflect total body iron stores and identify patients with iron overload syndromes or iron deficiency. Clinically, an elevated ferritin concentration can be seen not only in iron overload syndromes, but also caused by a wide variety of disparate conditions, including systemic inflammatory response syndrome (SIRS), infection, malignancy, and liver failure. Markedly elevated serum ferritin is typically thought to occur only in a few conditions, including adult onset Still’s disease (AOSD), macrophage activation syndrome (MAS), catastrophic antiphospholipid syndrome (cAPS), and the septic shock [4], which were included under a common syndrome named “hyperferritinemic syndrome” [5].

There is growing evidence that severe COVID-19, characterized by cytokine storm syndrome (CSS), can lead to acute respiratory distress syndrome (ARDS), as well as extrapulmonary multiple organ dysfunction, even death. Therefore, some scholars deduced that severe COVID-19 correlated with the “hyperferritinemic syndrome” [6–9], in which conditions, ferritin levels do not only reflect an acute inflammatory process response but rather may have a pathogenic role [5].

As a note, MAS is the alternative name for acquired hemophagocytic lymphohistiocytosis (HLH), with the clinical presentations including fever and splenomegaly, as well as cytopenias affecting ≥ two lineages, hypertriglyceridemia and/or hypofibrinogenemia, hemophagocytosis, hyperferritinemia, elevated soluble CD25 (sCD25), and impaired natural killer (NK) cell function [10]. Currently, HLH was included in the umbrella term of “hyperferritinemic syndromes” [5]. Therefore, as suggested by some investigators, HLH is worth drawing attention as a severe complication of COVID-19 [11–14].

Even though in the studies on COVID-19 patients, the levels of ferritin with other inflammatory markers have been reported [15–18], we rarely find dedicated researches in the works of literature investigating “hyperferritinemic syndromes” or “hyperferritinemia” in COVID-19 practice and giving detailed clinical characters and prognosis data in the periods. Therefore, the present study identified the prevalence of hyperferritinemia and the differences with non-hyperferritinemia from the perspective of COVID-19 at the pandemic outbreak.

## Materials and methods

### Study population and study design

A retrospective observational study [19] was further analyzed at the Tongji Hospital of the Hua Zhong University of Science and Technology in Wuhan, China. The Aid Hubei Medical Team of Peking University took over 295 patients from 8 February 2020 to 9 March 2020, together with Tongji Medical Team. This study (2020PHB080-01) has been approved by the ethics committee of the Peking University People’s Hospital, following the Strengthening the Reporting of Observational Studies in Epidemiology (STROBE) statement [20].

**Fig. 1:**
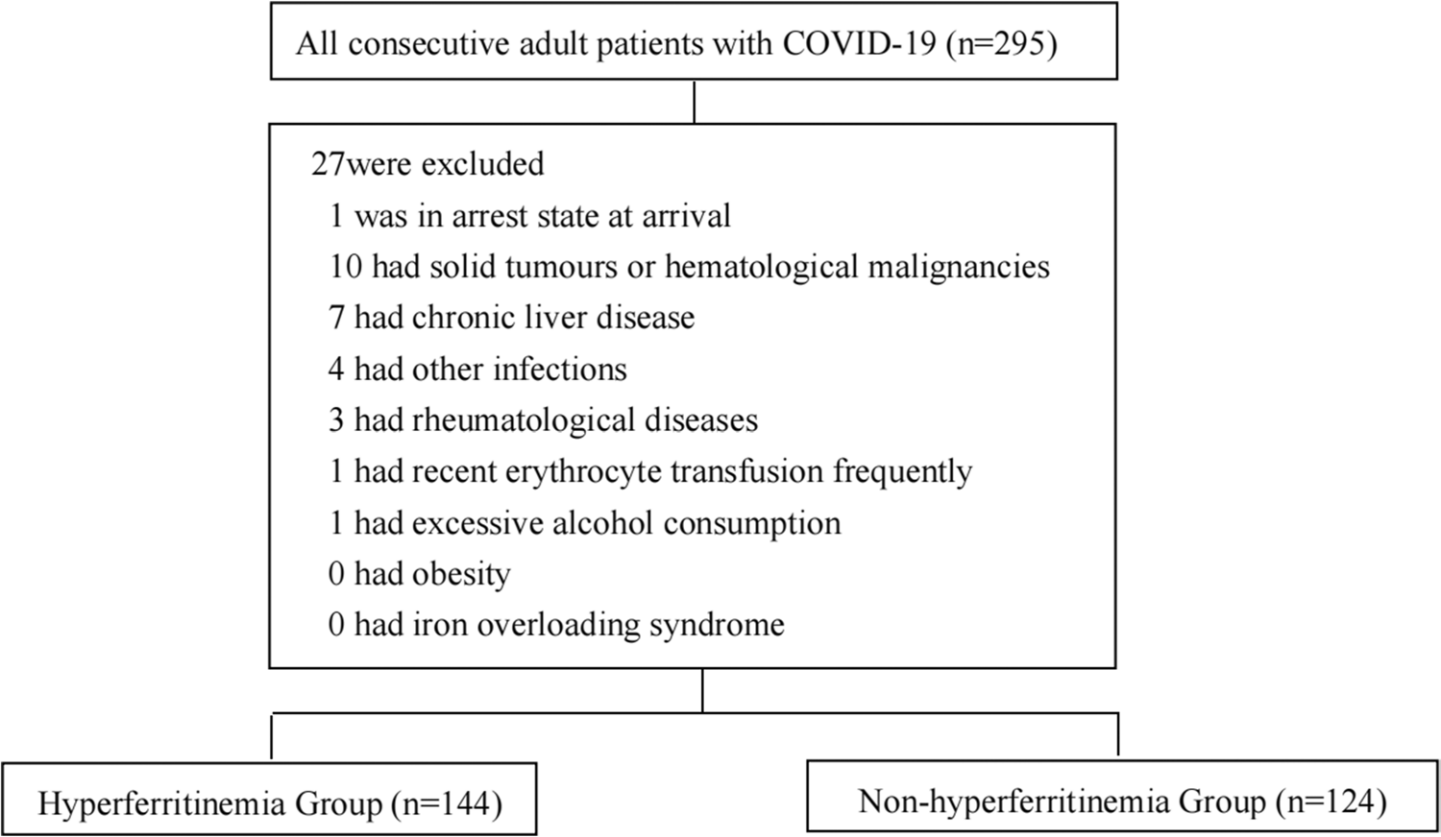
Flow chart of enrollment of study population

All the patients over 18 years old admitted and diagnosed with COVID-19 pneumonia [21] were screened. Patients in a state of arrest at arrival or who had recent erythrocyte transfusion frequently, chronic liver disease, excessive alcohol consumption, obesity, iron overloading syndrome, a solid tumor or hematological malignancies, rheumatological diseases, and other infections were excluded (Fig. 1). Patients were allocated into two subgroups based on the serum ferritin level: hyperferritinemia group (≥ 500 µg/L) and non-hyperferritinemia group (< 500 µg/L). The highest ferritin value was analyzed for the patients having more than one ferritin measurement of ≥ 500 µg/L.

### Data collection and definitions

Data were extracted from the standardized electric case report forms (eCRF). The data, including patient records and information, were anonymized and de-identified before analysis. The collected data included sex, age, temperature (T), neutrophil counts (N), platelet (Plt), hemoglobin (Hb), high sensitivity C-reactive protein (hsCRP), fibrinogen (FIB), D-dimer, triglyceride (TG), interleukin (IL)-6, in-patient duration, and complications, including ARDS, cardiovascular sequelae associated with COVID-19 (CSAC) [22], shock, coagulopathy, acute kidney injury (AKI), hepatopathy, and multiple organ dysfunction syndromes (MODS) [23]. All laboratory variables were recorded for 7 days after admission, as well as on every other day, and have been extracted at the same time with the maximum ferritin assessment. The definitions of terms related to HLH are shown in Table 1.

**Table 1:** The definitions of terms related to HLH

Criterion
1. Fever
T > 38.5
2. Cytopenias affecting ≥ 2 lineages
a. Hemoglobin < 90 g/L
b. Platelets < 100 × 10^9^/L
c. Neutrophils < 1.0 × 10^9^/L
3. Hypertriglyceridemia and/or hypofibrinogenemia
a. Triglycerides ≥ 3 mmol/L
b. Fibrinogen ≤ 1.50 g/L

### Statistical analysis

The data were analyzed with SPSS 25.0 (SPSS Inc., Chicago, IL, USA). The normality was tested through the Kolmogorov–Smirnov test. For describing variables, the median (interquartile range, IQR) and the non-parametric Mann–Whitney U test were adopted for continuous, *n* (%) and chi-square test for categorical. We assessed the risk of patients’ death based on hyperferritinemia and using the Kaplan–Meier survival analysis, followed by multivariable-adjusted Cox proportional hazard models adjusted for covariates and potential confounders. The area under the receiver operator characteristics (AUROC) curves analysis was used to determine the serum ferritin cutoff level for predicting the in-hospital mortality. *P* < 0.05 indicates that the difference was statistically significant.

## Results

### Comparison of hemophagocytosis between hyperferritinemia and non-hyperferritinemia

Among 268 patients, hyperferritinemia patients were 144 (53.73%), and non-hyperferritinemia patients were 124 (46.27%). Baseline characteristics of them were compared between the two groups (Table 2). There were 94 male patients (65.28%) in the hyperferritinemia group and 48 male patients (38.71%) in the non-hyperferritinemia group totally (*P* < 0.001). The hyperferritinemia group had a significantly higher median age than the non-hyperferritinemia group [67 years (IQR 57, 73), vs. 59 years (IQR 48, 68), *P* < 0.001].

**Table 2:** Demographics and clinical characteristics of hyperferritinemia syndrome

	All (*n* = 268)	Hyperferritinemia (*n* = 144)	Non-hyperferritinemia (*n* = 124)	*P* value
Demographics				
Male	142 (52.99%)	94 (65.28%)	48 (38.71%)	< 0.001*
Age (years)	64 (52, 71)	67 (57, 73)	59 (48, 68)	< 0.001*
Accompanied diseases				
Fever				
*n* (%)	36 (13.43%)	32 (22.22%)	4 (3.23%)	< 0.001*^C^
Median (IQR)	38.9 (38.6, 39.5)	38.8 (38.5, 39.5)	39.1 (38.9, 39.4)	0.254
Neutropenia				
*n* (%)	9 (3.36%)	5 (3.47%)	4 (3.23%)	0.911
Median (IQR)	0.68 (0.55, 0.86)	0.78 (0.40, 0.89)	0.66 (0.51, 0.85)	0.806
Anemia				
*n* (%)	18 (6.72%)	12 (8.33%)	6 (4.84%)	0.254
Median (IQR)	78 (63, 83)	79 (62, 84)	76 (63, 81)	0.542
Thrombocytopenia				
*n* (%)	32 (13.43%)	28 (19.44%)	4 (3.23%)	< 0.001*^C^
Median (IQR)	36 (21, 60)	36 (21, 55)	51 (20, 76)	0.648
Hypertriglyceridemia				
*n* (%)	16 (5.97%)	12 (8.33%)	4 (3.23%)	0.133^C^
Median (IQR)	4.52 (3.49, 6.02)	4.40 (3.23, 7.85)	4.52 (4.10, 5.50)	0.808
Hypofibrinogenemia				
*n* (%)	10 (3.73%)	8 (5.56%)	2 (1.61%)	0.169^C^
Median (IQR)	1.04 (0.81, 1.27)	0.60 (0.34, 0.56)	1.06 (0.98, 1.29)	0.036*
Comorbidity				
Hypertension	110 (41.04%)	67 (46.53%)	43 (34.68%)	0.049*
Diabetes mellitus	55 (20.52%)	37 (25.69%)	18 (14.52%)	0.024*
Cardiovascular disease	51 (19.03%)	32 (22.22%)	19 (15.32%)	0.151
Chronic pulmonary disease	23 (8.58%)	10 (6.94%)	13 (10.48%)	0.302
Chronic kidney disease	16 (5.97%)	8 (5.56%)	8 (6.45%)	0.758
Clinical outcomes				
In-hospital mortality	59 (22.01%)	54 (37.50%)	5 (4.03%)	< 0.001*
Mechanical ventilation	68 (25.37%)	64 (44.44%)	4 (3.23%)	< 0.001*^C^
In-patient duration	14 (8, 22)	17 (10, 24)	12 (6, 19)	< 0.001*

Data are presented as the median (IQR) or *n* (%). *P* was the comparison between hyperferritinemia and non-hyperferritinemia. *P < 0.05 was considered statistically significant

*LOS,* length of stay, *C* correct *P* value

In the hyperferritinemia group, fever (22.22%) was the most common among the symptoms, followed by thrombocytopenia (19.44%), anemia or hypertriglyceridemia (8.33%), hypofibrinogenemia (5.56%), and altered neutropenia (3.47%). Hyperferritinemia patients had a higher prevalence of fever (22.22% vs. 3.23%, *P* < 0.001) and thrombocytopenia (19.44% vs. 3.23%, *P* < 0.001) than non-hyperferritinemia patients. In addition, hyperferritinemia patients had significantly lower FIB level than non-hyperferritinemia patients [0.60 g/L (IQR 0.34, 0.56), vs. 1.06 g/L (IQR 0.98, 1.29), *P* = 0.036].

Of the 59 patients (22.01%) who died during hospitalization, 54 (37.50%) were hyperferritinemia patients, and 5 (4.03%) were non-hyperferritinemia patients (*P* < 0.001). The proportions of patients who required mechanical ventilator support were more significant in hyperferritinemia patients than non-hyperferritinemia patients (44.44% vs. 3.23%, *P* < 0.001).

An analysis of the comorbidities suggested that the prevalence of hypertension, diabetes mellitus, cardiovascular diseases, chronic pulmonary disease, and chronic kidney disease (CKD) among our patients was 41.04%, 20.52%, 19.03%, 8.58%, and 5.97%, respectively. Hyperferritinemia patients were higher than non-hyperferritinemia patients in accompany with hypertension and diabetes mellitus; two groups had obvious difference (46.53% vs. 12.62%, *P* = 0.049; 25.69% vs. 4.52%, *P* = 0.024).

From initial admission to death or release from the isolation treatment, the median 

duration was 14 days (IQR 8, 22). The median in-patient duration was statistically longer in hyperferritinemia patients compared with non-hyperferritinemia patients [(17 days (IQR 10, 24) vs. 12 days (IQR 6, 19), *P* < 0.001].

### Hyper-inflammation

**Fig. 2:**
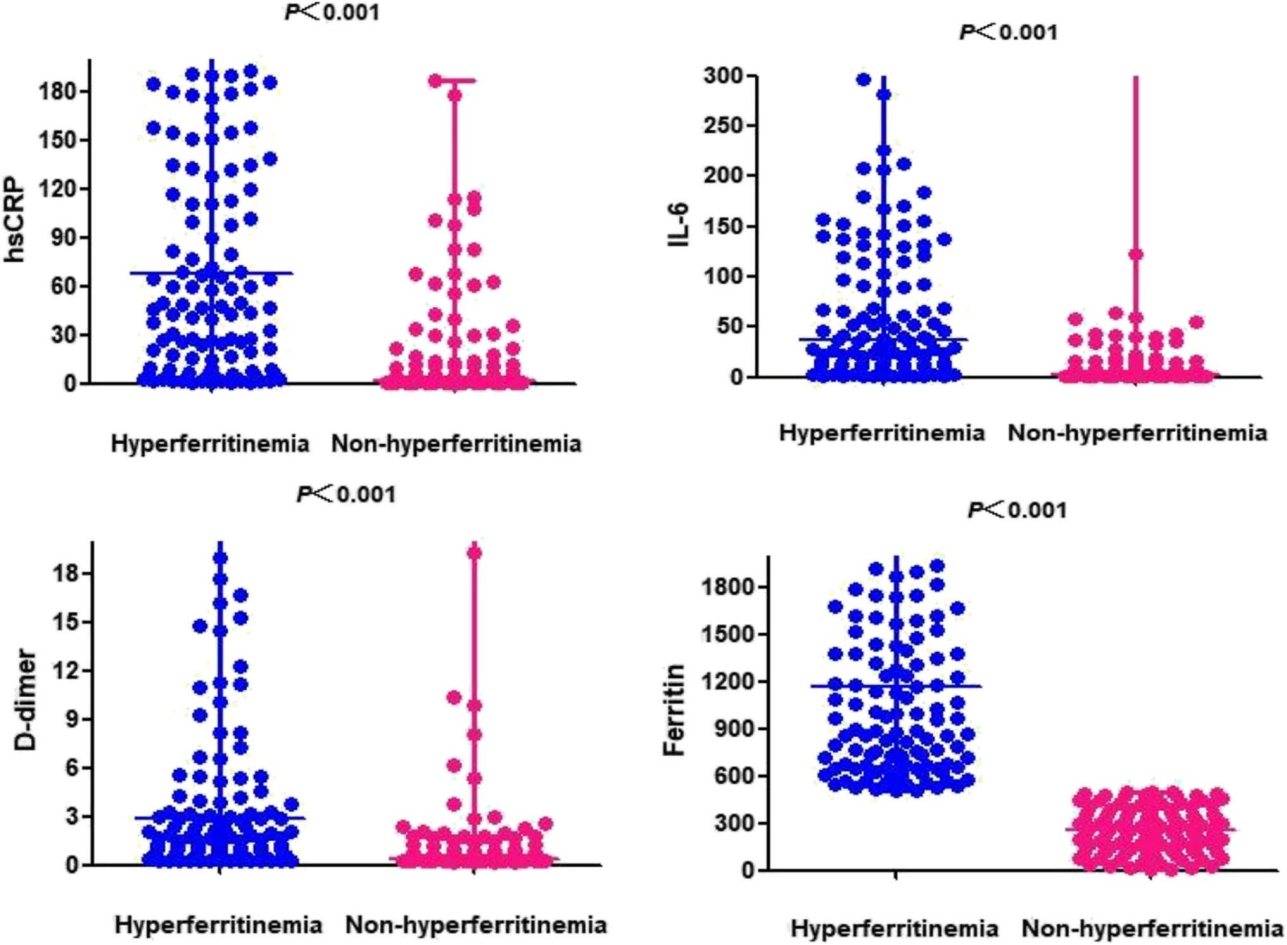
Inflammatory findings in the two groups

Figure 2 provided support that the dynamic changes of inflammatory factors were associated with hyperferritinemia. Table S1 summarized the results of cytokine storm description. The median level of IL-6 [37.25 pg/mL (IQR 10.58, 137.48) vs. 2.55 pg/mL (IQR 1.50, 8.95), *P* < 0.001], D-dimer [2.83 µg/mL (IQR 0.90, 16.53) vs. 0.41 µg/mL (IQR 0.22, 1.24), *P* < 0.001] and hsCRP [67.65 mg/L (IQR 18.25, 189.93) vs. 2.05 mg/L (IQR 11.45, 187.10), *P* < 0.001] was higher in hyperferritinemia patients compared to non-hyperferritinemia patients.

### Multiple organ dysfunction

**Fig. 3:**
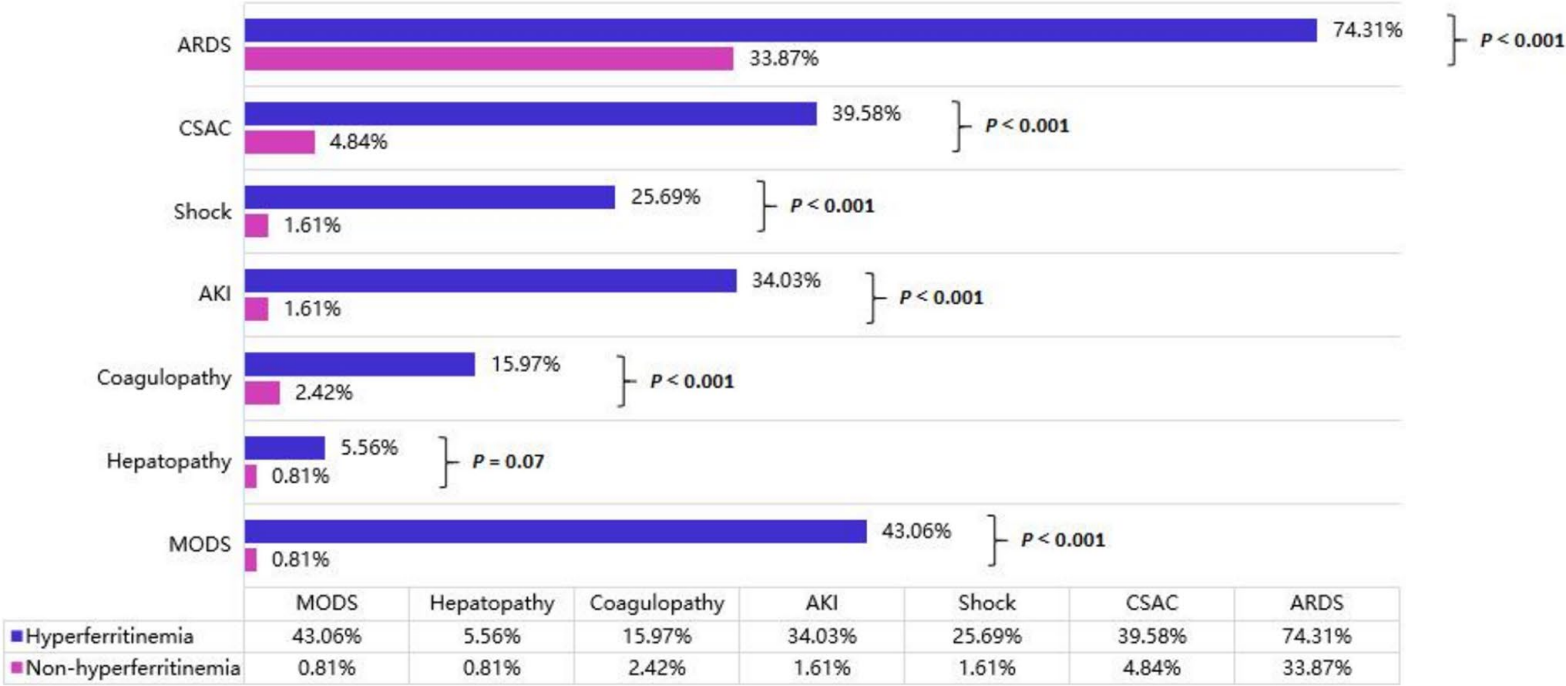
Spectrum of complications. AKI, acute kidney injury; ARDS, acute respiratory distress syndrome; CSAC, cardiovascular sequelae associated with COVID-19; MODS, multiple organ dysfunction syndromes

As Fig. 3 and Table S2 illustrated, during the hospitalization, 153 patients had complications, including ARDS (55.60%), CSAC (23.51%), shock (14.55%), AKI (19.03%), coagulopathy (9.70%), hepatopathy (3.36%), and MODS (23.51%). Among the hyperferritinemia patients, 74.31% showed ARDS, 39.58% had CSAC, 34.03% had AKI, 25.69% had shock, 15.97% had coagulopathy, 5.56% had hepatopathy, and 43.06% had MODS. Hyperferritinemia patients showed more complications compared to non-hyperferritinemia patients (*P* < 0.001), without any difference in hepatopathy rates of the two groups (5.56% vs. 0.81%, *P* = 0.07).

### Patients’ survival analyses

**Fig. 4:**
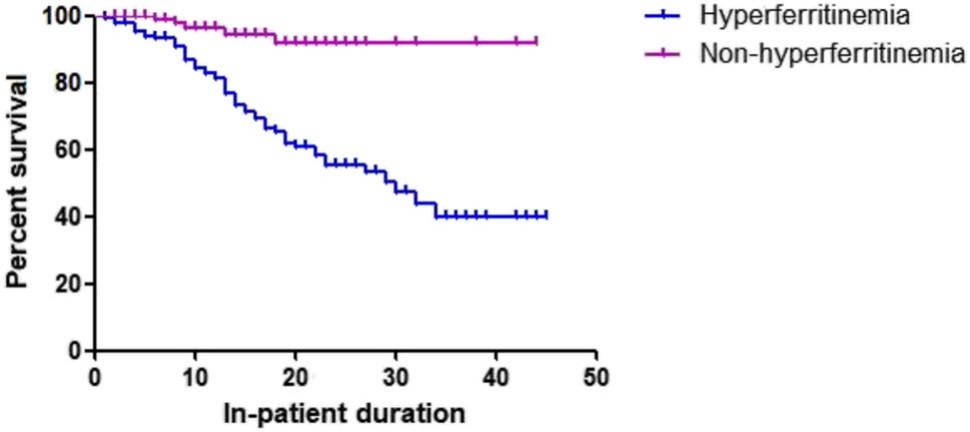
Cumulative survival according to serum ferritin level

Patients were followed up for dischargement from hospital after recovery. During this period, 54 cases in the hyperferritinemia group (37.50%) and 5 in the non-hyperferritinemia group (4.03%) lost their lives (*P* < 0.001). Figure 4 demonstrated that hyperferritinemia in Kaplan–Meier survival analysis significantly predicted poor prognosis of COVID-19 (log-rank test: *X*^2^ =21.278, *P* < 0.001).

In order to allow for the correction of potential confounding factors including age, gender, hypertension, diabetes mellitus, cardiovascular disease, chronic pulmonary disease, and CKD, we chose the multivariate Cox regression hazard model to test for differences in the hazard of death (Table 3). Age, hyperferritinemia, chronic pulmonary disease, and diabetes mellitus as covariates were found to be independently associated with in-hospital mortality from univariate Kaplan–Meier survival analysis. Age, chronic pulmonary disease, and hyperferritinemia were found to be significant independent predictors for in-hospital mortality [hazard ratio [HR] 1.041 (95% confidence interval [CI] 1.015–1.068), *P* = 0.002; HR 0.427 (95% CI 0.206–0.882), *P* = 0.022; HR 6.176 (95% CI 2.447–15.587), *P* < 0.001, respectively].

**Table 3:** Multivariate Cox regression hazard model for in-hospital mortality

	HR	95% CI	*P* value
Age	1.041	1.015–1.068	0.002*
Chronic pulmonary disease	0.427	0.206–0.882	0.022*
Diabetes mellitus	0.626	0.352–1.113	0.111
Hyperferritinemia	6.176	2.447–15.587	< 0.001*

*P < 0.05 was considered statistically significant

*CI* confidence interval, *HR* hazard ratio

### ROC curve analysis of the serum ferritin level for predicting the in-hospital mortality

**Fig. 5:**
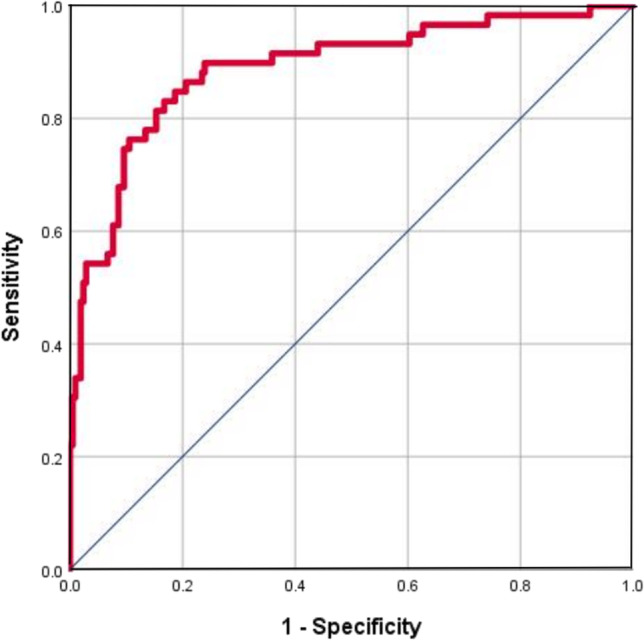
ROC curves of the serum ferritin level for predicting the in-hospital mortality

Figure 5 illustrates the ROC curves for the serum ferritin level for predicting the in-hospital mortality. The AUROC value of the serum ferritin level was 0.888 [95% confidence interval (CI), 0.836–0.940), *P* < 0.001], and the serum ferritin cutoff level was ≥ 971 µg/L based on the Youden index, with sensitivity of 0.831 and specificity of 0.833.

## Discussion

In the present study, the hyperferritinemia patients comprised 94 males (65.28%) with an average of 67 years (IQR 57–73), which is consistent with the previous COVID-19 research results [2, 24]; our study further confirmed this phenomenon. Moreover, our study further compared the difference between hyperferritinemia patients and non-hyperferritinemia patients.

One of the additional findings that are common in hyperferritinemia patients are fever, cytopenias, hypertriglyceridemia, and hypofibrinogenemia, and the symptoms overlap with HLH. Also, to our knowledge, a recent study showed that the optimal prediction accuracy for HLH is 4 instead of 5 conforming to HLH-2004 criteria [25]; these may explain why hyperferritinemia is highly predictive of mortality in COVID-19 patients. Despite regrettably that we could not consummate all of the laboratory tests, because sCD25 and NK cell activity were not readily available at the moment of a sudden outbreak of the epidemic, so we cannot diagnose HLH with existing guidelines [10], we were aware of the trend of their progressiveness towards the diagnosis of HLH. In HLH, high levels of pro- and anti-inflammatory cytokines and chemokines were generated from activated lymphocytes and macrophage secrete. IL and TNF**α** cause fever. Activated macrophages secreted ferritin, which generates plasminogen activator, which can lead to hyperfibrinolysis. Cytokines can inhibit lipoprotein lipase and hematopoiesis [26]. Hemophagocytosis may be not the only factor leading to the profound cytopenias, but also there are other pathophysiological mechanisms. In the review [27], the authors found that cytopenias are prominent among COVID-19, equivalent to one-third of patients. Moreover, the hypotheses are summarized as follows: First, the virus causes the apoptosis of hematopoietic and stromal cells in the bone marrow, thereby inhibiting hematopoiesis. Second, platelet consumption can be formed during microthrombus in disseminated intravascular coagulation (DIC). Finally, autoantibodies and complexes against antigens cause hemocytes to be destroyed by the reticuloendothelial system.

Other laboratory abnormalities, such as increased D-dimer, IL-6, and hs-CRP, were common among hyperferritinemia patients with COVID-19. They are all classical inflammatory markers and may spark a fatal CSS [28]. Unlike the other disease-associated CSS, incidences of COVID-19-associated CSS differ not only by gender and age, but also by ethnicity and comorbidities, etc. [3]. As has been shown above, although SARS-CoV-2 will affect both of the two kinds of genders, the CSS secondary to COVID-19 is more susceptible in elderly men, while the CSS associated with the other diseases mainly affected young people.

Based on our study, hyperferritinemia patients with COVID-19 showed more ARDS, CSAC, AKI, shock, and coagulopathy than non-hyperferritinemia patients, suggesting that hyperferritinemia can cause different degrees of damage from organ injury to death. Although hyperferritinemia is seen as a result of acute phase reactions usually, clinical interpretation of it often proves to be complex [29]. Accumulated data suggest that ferritin acts as a direct mediator between signaling molecules and the immune system, because the H subunit of ferritin plays a vital role in this mechanism [30].

Major strengths of our analyses were the utility of the serum ferritin level for predicting the in-hospital mortality in patients who were diagnosed with COVID-19 pneumonia. AUROC analysis of this biomarker yielded a value of 971 µg/L, which is considered to indicate an excellent predictive value.

The primary limitation of this retrospective study is that it was conducted in Wuhan during the early phase of the epidemic when the government quickly controlled it, so the study had limited sample sizes. Therefore, COVID-19-associated hyperferritinemia needs to be further confirmed in multicenter studies with larger sample sizes. Second, although we tried to completely exclude the factors closely related to hyperferritinemia and death according to previous literature reports [3–5], risk of confounding still remains, such as metabolic syndrome [31]. Although obesity was seen as an exclusive criteria, we did not exclude hypertension, cardiovascular disease, and diabetes mellitus. Therefore, in our study, the multivariable-adjusted Cox proportional hazard model analysis was performed to decrease the chance that biases will skew the results. Meanwhile, the patients in our study came from China’s main nationality, suggesting an underlying ethnic bias. Additional data from all over the world would provide a more comprehensive picture to better understand the results. Last, Schram reported that renal failure is the most common cause of elevated ferritin. However, as noted by Schram, the elevated ferritin occurring in renal failure patients is more close to secondary to other diseases, including infection, liver failure, and malignant tumors rather than renal failure itself [4], but completely ignoring this peculiar presentation may lead to bias caused by residual confounding. Fortunately, in our study, CKD was evenly distributed in the hyperferritinemia group and the non-hyperferritinemia group at the baseline, and CKD was not the independent risk factor for death in Kaplan–Meier survival analysis.

Taken as a whole, our results reveal several key points, some previously understood and others less apparent from prior research. Our primary contribution was to corroborate COVID-19-associated hyperferritinemia is the inflammatory state, with certain characteristics of HLH, which has been considered as one of the main causes for MODS and death. Indeed, due to the similarities in clinical manifestations and the high level of serum ferritin, severe COVID-19 was regarded as a fifth member of the “hyperferritinemic syndromes” [17]. Considered via this analysis, our results suggest that more attention should be paid to COVID-19 patients presented with hyperferritinemia syndrome, especially those with serum ferritin greater than 971 µg/L.

## Supplementary Information

Below is the link to the electronic supplementary material.

Supplementary file1 (DOCX 20 KB)

## Data Availability

For materials, correspondence and requests should be addressed to Z.J.H. and W.T.B.
