# Supplementary material for: Hemophagocytosis, hyper-inflammatory responses, and multiple organ damages in COVID-19-associated hyperferritinemia
Source: Ann Hematol. 2021 Dec 4;101(3):513–20. doi: 10.1007/s00277-021-04735-1 (PMC8643185; doi:10.1007/s00277-021-04735-1)
Supplement: Supplementary file 1 — Supplementary file1 (DOCX 20 KB) [file 277_2021_4735_MOESM1_ESM.docx]

**Table S1. Inflammatory cytokines of hyperferritinemic syndromes.**

|  | All  (n=268) | Hyperferritinemia  (n=144) | Non-hyperferritinemia  (n=124) | *P* value |
| --- | --- | --- | --- | --- |
| Ferritin (30~400µg/L) | 563 (269, 1234) | 1168 (728, 2100) | 254 (133, 359) | ＜0.001* |
| IL-6 (＜7.00pg/mL) | 11.85 (2.23, 52.68) | 37.25 (10.58, 137.48) | 2.55 (1.50, 8.95) | ＜0.001* |
| D-dimer (＜0.50µg/mL) | 1.14 (0.32, 4.21) | 2.83 (0.90, 16.53) | 0.41 (0.22, 1.24) | ＜0.001* |
| HsCRP (＜1.00mg/L) | 17.70 (1.83, 106.45) | 67.65 (18.25, 189.93) | 2.05 (11.45, 187.10) | ＜0.001* |

Data are presented as the median (IQR). *P* was the comparison between hyperferritinemia and non-hyperferritinemia. **P* < 0.05 was considered statistically significant.

hsCRP, high sensitivity C-reactive protein; IL, interleukin.

**Table S2. Organ dysfunction rates of hyperferritinemic syndromes.**

|  | All  (n=268) | Hyperferritinemia  (n=144) | Non-hyperferritinemia  (n=124) | *P* value |
| --- | --- | --- | --- | --- |
| ARDS | 149 (55.60%) | 107 (74.31%) | 42 (33.87%) | ＜0.001* |
| CSAC | 63 (23.51%) | 57 (39.58%) | 6 (4.84%) | ＜0.001* |
| Shock | 39 (14.55%) | 37 (25.69%) | 2 (1.61%) | ＜0.001*^C^ |
| AKI | 51 (19.03%) | 49 (34.03%) | 2 (1.61%) | ＜0.001*^C^ |
| Coagulopathy | 26 (9.70%) | 23 (15.97%) | 3 (2.42%) | ＜0.001*^C^ |
| Hepatopathy | 9 (3.36%) | 8 (5.56%) | 1 (0.81%) | 0.07^C^ |
| MODS | 63 (23.51%) | 62 (43.06%) | 1 (0.81%) | ＜0.001*^C^ |

Data are presented as n (%). *P* was the comparison between hyperferritinemia and non-hyperferritinemia. **P* < 0.05 was considered statistically significant. C,correct *P* value.

ARDS, acute respiratory distress syndrome; CSAC, cardiovascular sequelae associated with COVID-19; AKI, acute kidney injury; MODS, multiple organ dysfunction syndrome.
